# Bedside Politics and Precarious Care: New Directions of Inquiry in Critical Nursing Studies

**DOI:** 10.1097/ANS.0000000000000518

**Published:** 2023-11-20

**Authors:** Martijn Felder, Jitse Schuurmans, Nienke van Pijkeren, Syb Kuijper, Roland Bal, Iris Wallenburg

**Affiliations:** **Author Affiliations:** Erasmus School of Health Policy & Management, Erasmus University Rotterdam, Rotterdam, the Netherlands.

**Keywords:** bedside rationing, biopower, nursing, politics, precariousness

## Abstract

Health care systems are facing soaring workforce shortages, challenging their ability to secure timely access to good-quality care. In this context, nurses make difficult decisions about which patients to deliver care to, transfer to other providers, or strategically ignore. Yet, we still know little about how nurses engage in situated practices of bedside rationing. Building on the work of Giorgio Agamben and Judith Butler, we have developed a research agenda that homes in on a politics of bedside rationing. We argue that this agenda is essential to better understand the implications of scarcity for nursing and to explore new ways to cope with challenges faced.

THE COVID-19 pandemic has left its mark on the organization and delivery of health care services. Urgent health care demands, intense pressure on nursing staff, and limited resources forced services to be cancelled or postponed so that COVID-19 patients could be cared for.^1^ Consequently, timely access to other types of health care—such as elective surgery—became uncertain. Such uncertainty was widely accepted; there was common agreement that exceptional times required exceptional measures, especially because anyone infected ran the risk of a sudden and miserable death.^2^ Although the pressures caused by the pandemic have eased, many countries are still wrestling with enormous challenges, such as soaring workforce shortages, large volumes of catch-up care, and burgeoning waiting lists. They seem to have slipped into a new reality of scarcity that continues to undercut their ability to secure equal access to timely, good-quality care.

In dealing with these scarcity challenges, politicians and policymakers have called for more rationing of care. *Rationing* refers to

**Statements of Significance**

**What is known or assumed to be true about this topic?**

There is a burgeoning literature on workforce shortages amongst nurses in high- and middle-income countries. Yet, the issue is often taken at face value and first and foremost approached as a capacity problem. Hence, much emphasis is placed on developing strategies to make the nursing profession more attractive and to improve retention. Much less attention has however been paid to what current workforce shortages and other scarcity challenges mean for the nursing profession itself, in terms of how it changes the nature of everyday nursing work as well as the role and position of nurses in contemporary health care systems.

**What this article adds:**

We draw on a critical confrontation in the work of Giorgio Agamben and Judith Butler—as well as current developments in critical nursing studies more generally—to bundle and push forward an emergent direction of critical inquiry into nursing work in the context of scarcity. We particularly foreground a micro-politics of bedside rationing and develop a research agenda that addresses its political dimensions, practical implications, and ethical consequences. We posit that this agenda has important implications for nursing studies and nursing role development now that welfare states are becoming more provisional places.

practices of controlling and regulating the distribution of health care services in a context of limited resources.^3^ This policy response is not surprising; for several decades now, rationing has been central in discussions about the affordability of welfare states, often materializing in specific reimbursement packages and quality standards, informed by expert knowledge and scientific evidence (read: health technology assessments), and regulated through a plethora of state agencies and their regulatory instruments.^3^,^4^

Current workforce shortages have, however, turned rationing from a largely technical and bureaucratic endeavor into the mundane and urgent task of situated and everyday decision-making regarding who receives what care—and who does not.^3^,^5^ At stake is not only which kinds of care could be subjected to rationing—and how to do so fairly—but also how to understand and come to terms with practices of rationing taking place in daily care that goes beyond the scope of regulatory control. Examples are ad hoc cancellations of surgery, record-high waiting lists, postponed treatment, and rising nurse-patient ratios.^5^-^8^

Nurses play an important role in mundane rationing practices because of their key position in the organization and delivery of patient care.^1^,^9^ Weighing patient care needs against the backdrop of limited time and scarce resources, nurses must engage in different forms of prioritization, for example, in order to organize care and safeguard its continuation.^3^,^5^,^6^,^9^ This implies that they need to make difficult and situated decisions about which patients to spend time with, care for, transfer to others, put on a waiting list, or strategically ignore.^7^,^8^,^10^ Yet, we know little about how nurses engage in situated practices of priority setting and rationing.^7^ We furthermore have limited insight into the consequences of these practices, in terms of access to care, the quality of care delivered to different groups of patients,^11^,^12^ and the moral injury experienced by nurses who feel forced to make unwanted choices and find themselves dealing with the despair and anger of patients and family members who bear the brunt of these consequences.^13^,^14^

In this essay, we foreground the situated practices of priority setting and rationing that nurses engage in, as well as their consequences. Building on the work of Giorgio Agamben^15^-^17^ and Judith Butler,^18^-^22^ we approach such practices as political. We explore the different political dimensions of bedside rationing by referencing other articles published in this journal,^12^,^23^-^25^ as well as our own fieldwork in the Netherlands. Importantly, we do so without losing sight of the broader institutional contexts in which nurses—and the rationing practices they engage in—are embedded.^22^ We close this essay with a set of critical questions about bedside rationing as a basis for a research agenda, positing that this agenda is essential to (*a*) attune to a developing nursing epistemology sensitive to the societal, institutional, and systemic forces acting on the profession from different directions; and (*b*) allow nurses and nursing scholars to articulate academically informed professional responses to such forces.^26^

## SETTING THE SCENE: BEDSIDE RATIONING

The second half of the 20th century saw welfare states emerging across the Global North as governments sought to improve the economic and social well-being of their citizens. A particular point of concern was to establish equal access to health care and other social services. As medical knowledge and technologies advanced, more health problems were identified and turned into objects of care. This raised public expectations of what welfare states should deliver and, as a result, the demand for health care increased.

Caught between the aim of guaranteeing equal access to good-quality care and rising health care costs, welfare states sought to establish fair processes through which rationing decisions could be made.^27^ For example, they tried to structure and depoliticize rationing decisions by basing them on expert knowledge and scientific evidence (see Hauge and colleagues^4^ for a critical reading). In addition, a plethora of administrative agencies and regulatory instruments (eg, basic health care agreements and professional standards) were introduced to embed such rationing processes at different levels in health care systems.^28^ This layered approach not only made rationing an integral part of the functioning of many health care systems but also made it difficult to pinpoint which parties were responsible for rationing decisions, with differing administrative agencies, payers, and health care organizations struggling to interpret and shape rationing responsibilities administered to them *vis-à-vis* those administered to and enacted by others.

Today, attempts to establish systemic and fair processes for regulating the rationing of health care services are dovetailing with pressing shortages of nurses and other frontline workers. The situation is now one in which we have enough hospital beds and can harness impressive technological and medical advances, but no longer have enough hands available to care for all the patients and their needs, even when they are entitled to these services under existing legal agreements, professional standards, and expert assessment.^7^,^28^ In this context, nurses try to care as well as for as many people as possible, but with limited capacity and resources. What emerges is a form of invisible and implicit—but very real—rationing on nursing wards and behind people's front doors, performed by frontline workers, and materialized into everyday forms of priority setting in the distribution of time, attention, and resources.^8^,^10^

What strikes us is the lack of articulated responses among both nurses and nursing scholars about the bedside rationing nurses are engaged in and the politics implicated in such practices. In our fieldwork, at conferences, and during site visits, we hear nurses and their representative bodies articulating and worrying about workforce problems and their consequences for patient care. When it comes to public and academic debates, however, the issue is often taken at face value and either reified as a management problem that can be fixed through more efficient human resource management or depoliticized by redistributing the responsibility for rationing decisions to a plethora of administrative agencies and experts. Yet, it is nurses who face the soaring workforce shortages and how they impact patients, nursing peers, and health care delivery. It is therefore time for nurses to “speak truth to power” and join in discussions about policy responses to current scarcity issues, the value orientations underlying such responses, and the future of our welfare states.^26^ This requires a critical nursing epistemology and repertoire.^24^,^29^

## CURRENT DIRECTIONS OF INQUIRY IN CRITICAL NURSING STUDIES

In efforts to develop a more critical repertoire, nurse scholars are increasingly scrutinizing the agency of nurses in contemporary health care systems. They question some of the epistemic assumptions underlying the nursing literature, especially that nursing is about patient-centeredness and self-sacrifice.^9^ These scholars posit that nursing praxis would benefit from a more critical lens so as to grasp how and why nurses act the way they do and how such acts and their consequences should be interpreted and valued.^7^,^12^,^23^,^24^,^30^,^31^ In doing so, these scholars have tried to move beyond the widespread rhetoric that nursing is a power-deprived and apolitical profession in which individual patients take center stage—a rhetoric that more or less ignores the political environment in which nurses act.^30^

Critical nursing studies have started to reconsider nursing as a political entity within health care systems from several different angles.^23^,^24^,^31^ Some scholars insist that nurses are instruments of governmentality, responding to and in line with neoliberal state ideologies,^30^ and complicit in the responsibilization of patients and their families and the rationing of services.^32^ Others question such readings of nurses as instruments of a neoliberal regime and describe how nurses resist directive principles covertly in everyday care, for instance, by creating work-arounds^33^ or through other acts of subversion.^34^ Recent work on the role of nurses during the COVID-19 pandemic has moreover revealed nurses' key role in shaping the coordination programs that (for example) channeled the distribution of patients across hospitals while intervening in the distribution processes to make sure family members remained together.^1^ Others have reconstructed how nurses mobilized collective dissent toward macro-level institutional structures and managerialist ideologies forced on them.^31^

What is lacking within the realm of critical nursing studies, however, is a more theoretical approach that captures, understands, and articulates a politics of scarcity and bedside rationing and its implications for nursing practice. Because a politics of this kind is situated in everyday nursing practice—but simultaneously takes place in the broader context of health care systems—such an approach should capture the situated manifestations of bedside rationing and their implications for patients and nurses^7^ while remaining sensitive to the broader sociopolitical, organizational, and institutional contexts in which nursing work and beside rationing are embedded.^35^,^36^ In the following sections, we turn to the work of Giorgio Agamben and Judith Butler and discuss how they inform our inquiry into the politics of bedside rationing. We then focus on the theoretical differences between their approaches and the implications for critical nursing studies.

## GIORGIO AGAMBEN AND THE BIOPOLITICS OF EXCEPTION

There are interesting precursors supporting a conceptual pivot toward a politics of scarcity and bedside rationing in nursing. In this journal, for instance, critical nurse scholars have referenced the work of Giorgio Agamben^15^-^17^ to reveal the biopower implicated in the everyday organization and provision of care. The concept of biopower was coined by Michel Foucault^37^ to describe how modern governments seek to optimize the productivity of human life through a plethora of institutions and technologies. Agamben refined this concept by emphasizing the importance of sovereign power in ensuring that humans are protected by laws and have access to resources allowing them to live a productive and dignified life.^7^ In line with these ideas, nursing scholars have used the concept of biopower to underscore the “political thrust of activities that nurses take for granted in their daily practices (…) and how these shape the conditions of possibility for health, life, and death.”^24^^(p235)^ This has had important implications for how we conceptualize nursing work and scrutinize its consequences in times of scarcity. To show this, we first describe some of the basic tenets of Agamben's work and then relate it to nursing studies and vignettes from our own fieldwork.

A central question for Agamben^15^-^17^ is how within sociopolitical systems that place humanistic principles center stage—as is arguably the case in many welfare states—some human beings are marked and qualified as political and ethical subjects (eg, individuals with citizen rights, including voice, self-determination, and access to timely, good-quality care), whereas others are grouped into sociopolitical categories with an exceptional status in which these rights do not apply. Their fate is determined by those who do qualify as political subjects. Tracing this principle back to ancient Roman law, Agamben^15^ refers to this difference as a distinction between *bios* (qualified life—as in a person with political agency) and *zoe* (bare life—as in *homo sacer*, a person who is banned and could be killed by anybody, existing in the law as an exile from the law). According to Agamben, this distinction refers to a proto-political principle that concerns the bond between the sovereign, “with respect to whom all men are potentially *homines sacri*, and *homo sacer*, with respect to whom all men act as sovereigns.”^38^^(p257)^

Agamben argues that this proto-political principle is still very much ingrained in the extensive legal machineries of contemporary welfare states, one example being civil law protecting the rights of those who count as citizens while suspending such rights for those who do not. Moreover, this division can manifest itself arbitrarily in the political organization of welfare states in which, according to Agamben, scientists, physicians, economists, lawyers, and technocrats can all claim the throne of sovereign in specific situations and institutional contexts.^38^ Agamben's point is that, in the everyday ordering of social life, there are always those (often specific social groups) who can become categorized as “others” and consequently run the risk of having exceptional status imposed on them, from refugees losing citizen status to citizens with dementia losing the right to self-determination. This exceptional status reduces an individual's ability to claim rights or enjoy liberties while and potentially subjecting that individual to new orders of rule beyond the scope and protection of the law (or what is deemed fair or just) and therefore potentially inhuman in nature.^15^,^39^,^40^

### Logics of exception in nursing work

Nursing scholars drawing on the work of Agamben have shown how, in the coordination and delivery of care in nursing wards, logics of exception are crucial to mobilizing labor and resources—and hence to keeping things going, particularly when demand exceeds capacity.^12^,^23^,^24^ Espina and Narruhn,^12^ for instance, have revealed how in the United States, during the height of the COVID-19 pandemic, making exceptions became part and parcel of health care organizational responses. This manifested itself in ventilator allocation practices and decisions about scaling up and down patient care against the backdrop of limited health care staff and resources. On the one hand, decisions about ventilator use or scaling up care for individual patients were based on professional judgment and medical criteria such as age, life expectancy, and comorbidities. On the other hand, professional judgment and medical criteria were themselves significantly influenced by such social determinants as race, employment, and socioeconomic status. In allocating ventilators and making decisions about scaling up care for specific individuals (this pregnant woman, that young adolescent, the elderly obese [read the last one as categorical]), frontline workers reproduced disparities between specific social categories^12^—meaning that some patients had access to resources whereas others did not, sometimes with fatal consequences.

A vignette from our own fieldwork demonstrates a similar experience among elderly Dutch persons with early-stage dementia who became infected with COVID-19. It is the account of a health care manager, trained as a nurse, who volunteered to provide nursing care during the height of the pandemic. As she recalls:

In the Netherlands, during the first wave, specific COVID houses were established to care for patients discharged from hospitals but not well enough to go home. Patients who were too ill to stay home, but not ill enough to be hospitalized, were also brought there. They were all older persons, many with suspected or confirmed forms of dementia. They had all tested positive for COVID-19. One such COVID house had 36 patients. It was an old and shabby building without air-conditioning. It got very hot there in summer. At the beginning, 3 nurses tended to the patients during the day and 3 nurses at night. Later on, as the first wave ended, 9 patients remained, tended by 1 nurse per shift. In the words of one nurse, “the patients who remained were considered difficult cases. Current policy dictates that they need to stay there until they no longer test positive. But they all continue to test positive, partly because there are no isolation procedures within the building (rather, the building itself functions as an isolation site). All these patients have mild forms of dementia and the doctors have decided they cannot return home, but there is no room for them in nursing home eithers. The patients are becoming more and more agitated. They feel stuck and are anxious about the future. There are no daytime activities organized or television. Family is not allowed to visit. They fight with one another about petty things, such as the amount of raisin bread someone ate or a lost slipper. Because I (the nurse) am alone during shifts, there is no way that I can tend to their physical and emotional needs. I can't even go to the toilet.” (Derived from a diary study among Dutch nurses conducted in the spring and early summer of 2020)

Quarantining older and sick people in an improvised building without allowing them to make their own decisions—or receive the care that they need—comes close to what Agamben has identified as the danger of violence inherent to states of exception and the associated suspension of civil rights and liberties in times of crisis.^17^ At the intersection between crisis management and health care provision—when patients are most vulnerable and professionals are under pressure—there is an extreme risk that collective norms will be abandoned, voices silenced, and the quality of some human lives, such as elderly persons with early-stage dementia, forfeited to protect those of others.^7^,^23^

### The gradual reordering of nursing principles

Agamben^17^ warns us that once society has entered crisis mode, there is a strong possibility that the suspension of norms and laws will become an ongoing, prolonged situation, slowly incorporating more groups and social categories. In this line of thinking, exceptional states can be imposed on entire populations, with a gradual reordering of governance principles and the value orientations that drive them.^17^,^40^

An important example of this is the normalization of workforce shortages in the wake of the COVID-19 pandemic and its implications for the organization and provision of health care.^41^ The following vignette from our fieldwork illustrates how such normalization can indeed go hand in hand with the establishment of new nursing roles and renegotiation about what is at stake in terms of the care provided in particular situations. The district nurse who features in the vignette represents a new role introduced in rural areas where health care organizations struggle with staffing capacity.^10^ District nurses work across organizational boundaries and safeguard the continuation of care trajectories when staffing levels are low (especially weekends and nights). As such, district nurses help individual health care organizations ensure the continuity of patient care and turn the challenge of doing so into a shared responsibility. In this context, district nurses must respond creatively to a plethora of care needs that can arise unexpectedly.

The attending physician at the emergency post received a phone call from an older woman (aged 80) who had just visited her sister and was concerned about her confused behavior. The caller was too ill and frail to take care of her sister that weekend. The physician decided to contact the district nurse and ask him to visit the sister, as she was receiving home care. The district nurse was pressed for time because his shift was quickly filling up with clients at distant locations needing care (eg, leaking catheters; bandage changes; falls). To assess the situation, the district nurse and physician looked more closely at the woman's patient record and concluded that it was a long-term housing problem and therefore, in their view, a responsibility that lay first and foremost with the municipality. As long as her confused behavior was not immediately harmful to her or her environment, it was not urgent. They decided to postpone until Monday, when the patient's own physician would be available and municipal workers were back in the office. In the meantime, responsibility for monitoring the situation was passed back to the woman's sister. (Derived from fieldwork among Dutch district nurses in the spring of 2021)

On the job, district nurses engage in distributed forms of priority setting as they carefully negotiate what is at stake in a particular situation (in the aforementioned example, if no one is harmed, then a confused state does not require urgent action) and who is responsible for dealing with it and when (in this case, the municipality and the patient's own physician next week, rather than the attending physician or district nurse over the weekend). This means that understaffing not only provides the reason for introducing district nurses—because it is a problem they can mitigate—but also becomes an issue that district nurses themselves deal with as part of their own “daily” practice. District nurses therefore not only manifest themselves as “gap-fillers” and “creative problem-solvers” but also become experts in triage, defining the boundaries of their responsibility along the way.^10^ In this light, the older woman's entitlement to receive care through different arrangements (long-term care arranged through municipalities and basic care paid by health insurers and provided by nurses) did not work to her advantage. In fact, her entitlement to these different arrangements made it possible for the district nurse to deflect responsibility to the municipality. Importantly, this was enabled (and legitimized) by an institutionally fragmented health care system in which entitlement to care is granted and protected through different juridico-political arrangements and the scope of funding schemes and professionals they encompass.

In the next section, we unpack the consequences of multiple—and therefore partial—juridico-political arrangements, as well as the fragmented health care systems from which they stem. For now, we argue that Agamben-informed analyses typically show that nursing is not always and not only about connecting to patients, recognizing their needs, and tending to them in meaningful ways (as Tronto^42^ stated in *An Ethic of Care*). Instead, it is also about dissociation, disparity, and exclusion, especially when nurses try to make things work for a majority of their patients, sometimes at the expense of a few. This is arguably a natural foundation of nurses' political and ethical practices,^24^ but we also argue that it becomes more visible and stringent in times of scarcity and rationing, such as during the COVID-19 pandemic^12^ or the current workforce shortages plaguing many welfare states.^41^ Rationing and prioritization in everyday nursing practice hence raise important questions about how nurses decide whose interests will prevail and whose lives—or, more generally, whose quality of life—will be suspended so that they can tend others. Answering such questions also means homing in on the situated and relational circumstances in which nurses engage in the organization and provision of care, set priorities, and make rationing decisions.^9^ Yet, it is here that Agamben's political principles become too static to aid interpretation and additional scholarly work is necessary. We do this by turning to the work of Judith Butler.^18^-^22^

## JUDITH BUTLER AND THE SITUATED POLITICS OF PRECARIOUS LIFE

In *Who Sings the Nation State*, Butler and Spivak^22^ argue that the relative protection that citizens of welfare states enjoy is the product of a complex web of juridico-political arrangements, technical-material infrastructures, geostrategic positions, and historically situated institutions and valuation regimes. Each of these inscribe into a situation that can act how and in relation to whom in specific, sometimes contradictory ways.^43^ This means that legal protections and their suspension are always partial and do not stem from any specific center of power that decides sovereignly over the status of individual patients. Entitlements to health care can be established and suspended in different ways, on different grounds, by different health care providers, and in different situations. It is therefore impossible to assume that legal protections and entitlements are intact prior to their suspension, as Agamben-informed analysis would posit in interpreting something as an exception or someone as acting sovereign.^38^ An important example is the adverse consequence of the various juridico-political arrangements to which the older woman in the previous vignette was entitled and how she therefore “fell between the cracks,” particularly so because health care providers use such arrangements to deflect responsibility and shift it to others.^7^

Butler and Spivak's^22^ polycentric reading of the organization of welfare states allows us to foreground that physicians and nurses not only interact with patients but must also comply with professional standards and can be called to account for their decisions and actions (and the quality and safety of care provided) by health care inspectorates and disciplinary boards. Health care inspectorates, in turn, must adapt their quality and safety frameworks to societal changes, advances in medical knowledge, and professional practice while being under permanent public scrutiny.^44^ Where market mechanisms have been introduced (as they have been in the Netherlands), each of these actors must furthermore deal with—or at least take into account—market dynamics, which are, in turn, monitored and restricted by yet another group of state authorities, and so forth.^43^ As the following vignette demonstrates, there can indeed be an overabundance of arrangements, infrastructures, institutions, and valuation regimes idiosyncratically tethered together in a specific situation in which care is established or reestablished for a patient. We illustrate this with another excerpt from our observations.

Today, I'm shadowing Elise during her daytime shift on a surgical ward in a regional Dutch hospital. She is participating in a pilot study organized by the hospital in which nurses are assigned a distinct coordinating role among their peers. While we tend 2 patients during the morning round, she explains that her new role means having a helicopter view of the ward, coaching coworkers where necessary and assuming leadership when needed, to ensure that health care trajectories on the ward are aligned. Because she finishes tending her own patients sooner than expected—a rare occurrence in everyday nursing—Elise has some spare time. She decides to read up on one of the patient cases, a 93-year-old woman who fell in the shower 3 weeks ago and has been hospitalized ever since. The physicians say she has recovered and is ready to go home, but her nurses think she should not because her husband is unable to care for her and they suspect she has a mild form of dementia (as recorded in her electronic patient file). The woman is becoming increasingly demanding (needing additional psychosocial and bodily care), and the nurses are a bit agitated about her as they struggle to juggle her demands and caring for other patients. The patient is furthermore occupying a hospital bed on the surgical ward while the waiting list for surgery is growing and health insurers are pressuring hospitals to use their bed capacity more appropriately (read: not provide care that should be provided elsewhere via long-term arrangements). Elise's coworkers have recently suggested transferring the patient to a nursing home, but neither she nor her husband or son want this. Elise decides to dedicate time to solving this problem in her role as coordinating nurse. She contacts a nursing home downtown and manages to arrange a spot despite the waiting list. At the same time, she convinces a medical resident to inform the family that the patient is being moved to that nursing home. She tells the patient, kindly but firmly, what will happen. The patient has, however, just been instructed by her son—who was informed by the medical resident by phone about the plan—not to consent and to wait until he arrives to sort things out. With panic in her eyes, she says “no” and “wait” and holds on to the side of a table. Elise presses ahead and calls an ambulance to take the patient to the nursing home. The ambulance arrives 20 minutes later. In the meantime, the patient has resisted every effort to arrange for her departure, hoping to buy time for her son to arrive. As soon as she sees the ambulance staff entering, she starts panicking. Elise and the ambulance staff talk about how to proceed and decide to restrain her on a stretcher and roll her out of her room. The patient cries and continues saying “no” as she is rolled to the elevator, her objections slowly fading as the swing doors close. Elise looks at me, seemingly moved by what just happened. I share her confusion and feel implicated. (Derived from ethnographic fieldwork among Dutch nurses in the spring of 2022)

In this vignette, the objectives of an organizational pilot study, a coordinating nurse's spare time, the nursing team's assessment of patient needs, a suspected case of dementia, constant pressure to free up hospital beds, and even agreements with health insurers about bed allocation policies appear to coalesce into a situation in which an individual patient's wishes and right to consent—a patient suspected of having dementia and making increasing demands on nursing peers—were momentarily suspended. It was a critical moment, marking a shift from the prospect of returning home to her husband to living in a nursing home on her own.

This example echoes an observation by Waring and Bishop,^7^ who studied untimely hospital discharges and revealed how in increasingly complex health care systems—in which multiple disciplines and resources need to be aligned around the treatment of individual patients—professional, sociocultural, and organizational arrangements lead to certain lives being considered less valuable than others and therefore more amenable to harmful treatment. Importantly, the states of exception emerging in their study were not necessarily the consequence of a specific crisis situation, individual decision, or legal determination. Instead, they were an inadvertent outcome of incompatible and partial decisions in an overly fragmented organization of health care services. They resulted from a complex web of power relations in which the reasons to act, provide care, and take responsibility were vastly and idiosyncratically distributed.^7^

When comparing Waring and Bishop's^7^ interpretation of untimely hospital discharges with the Agamben-informed studies discussed in the previous subsection, 2 things stand out. First, the outcome is by and large the same: the rights of some patients are momentarily suspended to get things done for others. Second, the 2 analyses offer different explanations for why and how this happens. While Agamben-informed analysis tends to foreground sovereign acts of decision-making or legal determinations, both Waring and Bishop^7^ and Butler and Spivak^22^ point toward a complex and polycentric health care ecosystem in which responsibilities to act and provide care are distributed and therefore always partial. This raises important follow-up questions about why such responsibilities are deflected or shifted elsewhere in some situations (as in the case of the district nurse) whereas in others they are embraced. Butler^18^,^20^ has therefore insisted that more specific, situated, and relational approaches are needed to understand how some patients come to suffer from failing juridico-political arrangements and professional networks of support whereas others do not.

### A more relational approach

In Agamben-informed nursing studies, those accorded exceptional status are approached as passive victims of biopower, voiceless, helpless, and mercifully subjected to the goodwill of nurse professionals who exert sovereign power over them in specific situations. That nurses can make a difference—both positive and negative—in the care received by individual patients has long been recognized in the nursing literature.^25^ Nursing scholars have therefore sought to identify (implicit) nurse biases toward specific groups of patients—such as ethnic minorities or groups with stigmatizing diagnoses—and to reflect on them in nurse training programs to reduce their impact on nurse-patient interactions.^45^

While Butler^20^ undoubtedly agrees about the presence of (implicit) biases among nurses, she also cautions not to overcommit to a radically asymmetrical ethico-political conceptualization of the relationship between, in our case, nurses and their patients, that is, a conceptual relationship in which the altruistic and caring, yet also (implicitly) biased and politically proficient nurse subject is positioned as sovereign over the apolitical patient subject.^38^ Not only does this oversimplify the complex distribution of power in health care practices and the many juridico-political arrangements and actors involved but it also distorts the experiences, needs, and actions of patients and nurses as they interact and together try to figure out how to respond to care needs.^23^ Let us look at another example from our fieldwork that foregrounds such interactions.

An elderly patient on a neurology ward is deteriorating quickly and there is not much more that the physicians and nurses can do to improve his condition. After talking to the patient, who has indicated that he does not want to be resuscitated if something happens, they decide with him to switch to palliative care. There are, however, some challenges in organizing the palliative care trajectory in a way that the nurses consider appropriate. The patient is in an isolation room because he tested positive for COVID-19 when he entered the hospital 2 weeks ago. This has serious consequences for the time and attention that nurses can give him because they need to put on protective aprons and masks every time they enter the room. Even worse is that the patient has largely lost contact with his family. The nurses have gathered from family members that he is a difficult man and that most of them have cut off all contact with him. The nurses themselves have a very different experience of the patient, however. He is polite, kind, and up for a short conversation, even under the current circumstances. The nurses feel very unhappy that the patient is spending his final days alone and in isolation. They have started putting pressure on the physicians to at least cancel the patient's COVID-19 status. This would make it much easier for the nurses to visit the patient and spend some time with him. Two days ago, the physician on call refused to do that because the patient still had a cough. It was against protocol, he argued. This angered the nurses because it would mean that the patient needed to remain in isolation until he passed away, as there was little chance that the cough would disappear. They also questioned the association between the cough and the COVID-19 infection, while aware that there was no way to disprove this association because the patient would continue to test positive for a while. Today, however, a different, “more nuanced” physician is on the ward and the nurses pressure him to change the patient's status. In the meantime, they are already ignoring the most stringent isolation rules, keeping the door ajar, entering more frequently to check on the patient and having brief conversations. (Derived from ethnographic fieldwork among Dutch nurses in the fall of 2022)

In this vignette, nurses feel unhappy about an older male patient spending his final days alone and in isolation. He is a patient the nurses can easily relate to—and empathize with—because he is kind, polite, and up for a conversation. The connection they feel lead them to stand up for him (against the physician's reading of the situation) and engage in minor acts of subversion (ignoring some of the more stringent protocols). They do this to deliver the palliative care they consider appropriate. The vignette featuring the elderly woman who was becoming increasingly demanding and agitated shows that things can also turn out very differently, however. In that case, nurses followed bed allocation policies to the letter to transfer the patient elsewhere.

One could argue that the nurses in these examples were (implicitly) biased against the elderly, demanding woman with suspected dementia. This argument, however, could very easily reduce nurses' idiosyncratic actions of solidarity to a single judgment call that they made sovereignly—based on the woman's suspected diagnosis—and background the complex relational, organizational, and institutional context in which nurses connect to patients, patients connect to nurses, and nurse-patient relationships are established and reestablished continuously.^20^,^38^ Everyday examples from our vignettes include the following: the social capital that the elderly male patient has accrued—and invested—in connecting with the nurses, as opposed to the demanding elderly woman (ie, patients are not just passive receivers of care); the opportunity that arises when another, “more nuanced” physician oversees the ward and “might” be moved to change the patient's status (relationships are established in broader networks of actors); and the legal arrangements, bed allocation policies, and quarantine rules that were either followed, ignored, or bended to provide the care that nurses deemed convenient, appropriate, or fair (there are infrastructures to be taken into account [see previous section]). Informed by Butler, we therefore challenge the argument of an asymmetrical ethico-political conceptualization of the relationship between nurses and their patients and propose instead that it is not *only* nurses' empathy and bias that define the nurse-patient relationship.^11^

Butler^20^,^21^ urges nursing scholars to focus on the more situated and relational dimensions through which care is continuously established and reestablished in everyday care delivery. She furthermore insists that such relationships and engagement are informed and structured by systemic conditions, such as the juridico-political arrangements, technical-material infrastructures, geostrategic positions, and historically situated institutions and valuation regimes we mentioned earlier.^7^,^22^ To foreground this interplay between systemic conditions, relational engagement, and their idiosyncratic outcomes, Butler^18^,^19^ introduces the concept of precariousness. She uses this to draw our attention to the specific relationships, practices, and conditions through which certain patients suffer from failing juridico-political arrangements and social and economic networks of support—potentially exposing them to injury, violence, and death—whereas others are not.

## THEORETICAL DIFFERENCES WITH POLITICAL IMPLICATIONS

According to Butler,^20^ whatever counts as timely access to good-quality care—or, in the case of the last vignette earlier, a humane death—should be explored with situated specificity rather than by referring to Agamben's abstract and timeless legal machineries, such as the states of exception and sovereignty central to his work.^38^ Importantly and in this context, timely access to good-quality care is considered an idiosyncratic accomplishment rather than an a priori entitlement (from which a person or social category can arguably be excluded). This is the case even where we have sought to guarantee equal access to health care services through the sort of regulatory measures present in the bureaucratic frameworks of contemporary welfare states. We argue that precariousness is particularly relevant now that the traditional welfare state is becoming a more provisional place in the face of scarcity politics, workforce shortages, institutional fragmentation, and a dilution of rationing practices.^22^

Butler's critique of Agamben is more than just a call for more specificity, however. In her writings, Butler^18^-^20^ refers to a folding of vulnerability and agency, instead of a polarity between *bios* and *zoe*, so as to capture—in our case—the everyday and negotiated ordering and provision of health care services.^38^ Such an approach calls for a new conceptualization of the political subjectivity of nurses and has important consequences for their normative responsibilities and political actions in times of scarcity and rationing.^19^,^46^,^47^ Agamben-informed nursing studies would prompt nurses to speak up and problematize the consequences of bedside rationing for specific social categories—consequences that subsequently need to be addressed on higher political levels through public debates and more inclusive policies.^23^,^24^ Butler, on the other hand, would prompt nurses also to recognize and embrace the responsibility to continue to act and intervene in the complex ecosystems in which patient care is continuously established and reestablished.

Butler's position can be read as a call to continue noting the longing, suffering, and vulnerability of others and, importantly, to feel the responsibility to act on that observation. In our fieldwork, however, we also noticed that workforce shortages drive nurses toward feeling not unmoved but rather overwhelmed by the many forms of longing and suffering they encounter in daily practice. This forces them to prioritize certain patients or to ration their responses in order to tend to the needs of the many. The very bonds between nurses and their patients—so often celebrated as part of the core identity of the nursing profession—may therefore also lead to moral injury (as in someone realizing that they are unable to properly address the longing and suffering of another) and even a withdrawal from the profession rather than activation or reactivation (see also Ruti^48^ for a critical discussion of the flipside of Butler's relational ontology and ethico-political position). In turn, Lorey^49^ recognizes in the precariousness of patients—and the nurses who relate to them—a potential political power that urges us to rethink nurse-patient relationships and prompts us to explore new modes of caring and organizing care.

The way in which we study, conceptualize, and position the political subjectivity of nurses is therefore not without its consequences. On the one hand, it could signify and support an important repositioning and revaluation of the key role of nurses in contemporary welfare states,^1^ which urgently need to develop more adequate responses to pressing scarcity issues. On the other hand, it could lead to a situation in which problems that should be addressed on systemic levels become (re)politicized as nursing problems, or nurses' responsibilities, that subsequently drive out the very professionals so desperately needed to safeguard health care delivery in times of scarcity. One way or another, we still have very little insight into how bedside rationing is actually practiced in the context of scarcity and how this impacts the nursing profession and individual nurses and patients. The vignettes presented earlier can be considered a first step in that direction.

## TOWARD A RESEARCH AGENDA FOR CRITICAL NURSING STUDIES ON BEDSIDE RATIONING

It is not our intention in this essay to choose between Judith Butler and Giorgio Agamben or to occupy a moral high ground from which to reflect normatively on nursing practice. Instead, our intention is to develop a theoretically informed critical research agenda that homes in on a politics of bedside rationing and is able to scrutinize its consequences. In this light, the concept of precariousness allows us to foreground that good care is a situated and relational accomplishment rather than the product of a proto-political condition or legal machinery. It furthermore draws attention to the interdependencies between patients and nurses, as well as the latter's ability and inability to recognize and respond to the needs of the former within health care systems faced with scarcity challenges and in which the responsibility to organize and provide care—as well as assess its fit and quality—is vastly and idiosyncratically distributed. Agamben-informed nursing work, however, also continues to sensitize us to potential structural disparities among specific groups of patients, influenced by social determinants such as race, employment, socioeconomic status, or specific health conditions.^7^,^11^,^12^ Indeed, in our vignettes, elderly patients suspected of having dementia were particularly vulnerable to ending up in a precarious situation, especially when there were partial arrangements and distributed responsibilities in periods of scarcity and irrepressible rationing.^12^,^17^

Sensitized by the work of Giorgio Agamben and Judith Butler, we argue that nursing scholarship must home in on a politics of scarcity and bedside rationing without losing sight of the institutional contexts in which nurses—and their patients—are embedded. By illuminating everyday acts of looking and looking away, planning and improvising, aiding and ignoring, hiding and standing up—and by approaching these as energetic, relational, and intentional—we can reestablish nurse agency as a key element in safeguarding health care provision in times of scarcity and the associated bedside rationing practices. By simultaneously staying sensitive to the historical and institutional conditions that inform and enable such nursing acts, we can scrutinize what counts in specific situations as appropriate care. In the following text, we present a set of empirically, theoretically, and politically informed questions as the basis for a research agenda.

### On the everyday politics of bedside rationing by nurses and its consequences

How do nurses practice priority setting and bedside rationing in times of scarcity? What kind of new interdependencies emerge between patients and nurses (*vis-à-vis* other health care actors involved)? How does precariousness emerge in the process? How do nurses and health care organizations detect and respond to such precariousness (or not)? How can we understand and respond to the moral injuries implied in bedside rationing and the consequences for individual nurses and the nursing profession?

### On the polycentric and layered institutional context in which bedside rationing unfolds

Which legal (and institutional) frameworks are in place to channel and protect timely access to quality care and which actors are involved in enacting these arrangements (both at the policy level and in everyday practice)? Which cracks emerge between the different juridico-political arrangements and the polycentric and partial distribution of rights and responsibilities in extant health care systems? Which patients and social categories are vulnerable to failing juridico-political arrangements and social and economic networks of support?

### On the professional and ethical implications of bedside rationing in a polycentric and layered context

What does this polycentric and layered institutional and juridico-political context mean for nursing practice and nursing ethics? What counts as good nursing practice in times of scarcity and which new valuation and accountability regimes emerge from this? Which roles do professional associations, health care regulators, and policymakers play in that process and how do nurses themselves contribute to such processes, for example, in discussions about good nursing practice in times of scarcity?

### On political advocacy and the (re)politicization of bedside rationing by nurses

Which roles do nurses play in (re)politicizing bedside rationing and its consequences? What knowledge and skills do nurses need to take on such roles? How can nurses take up a position in health care organizations—and policymaking more generally—that ensures that their voices are heard and influence decision-making? How can the (critical) nursing sciences—as well as professional education and development—equip nurses with the tools to raise their voices, share their experiences, mobilize, and act?

### On comparative analysis between countries

How can we understand differences between countries in how their health care systems are organized, scarcity challenges present themselves, and nurses face such challenges? Which differences do we see in how the nursing profession responds to such challenges and what can we learn from these differences? How can the critical nursing movements emerging in various countries learn from and support one another?

## CONCLUSION

To address the political dimensions, practical implications, and ethical consequences of bedside rationing, more critical nursing scholarship is warranted. Such scholarship should foreground how nurses muddle through, deal (and struggle) with, and position themselves in relation to scarcity issues and the uncertainties associated with them.^23^ It should, moreover, thematize and theorize the shifting foundations of nurses' political and ethical practices in times of scarcity^12^ and explore whether and how nurses can (or should) use political advocacy to discuss and (re)politicize bedside rationing and its consequences, opening it up to public scrutiny.^26^ To support this, we have drawn on the work of Giorgio Agamben and Judith Butler and related their work to current developments in nursing studies so as to concentrate and advance an emerging avenue of inquiry in critical nursing studies.

The research agenda we propose is not one that necessarily reinvigorates an image of the altruistic and caring nurse and protects it from an Agambean reading of nursing as inherently violent (as previously suggested in this journal^23^). Instead, we think our agenda could produce much more nuanced and specific insights—both illuminating and dark—into the dilemmas that nurses face when confronted with irrepressible rationing and needing to engage in mundane forms of priority setting in the distribution of their time, attention, and resources. It should furthermore help problematize the depoliticization of such rationing as a nursing organizational problem that can be fixed through better or more efficient human resource management. In doing so, it opens up both the practices and consequences of bedside rationing to public scrutiny and may lead to new modes of conceptualizing, organizing, and positioning nursing work, now that the welfare state is quickly becoming a more provisional place.
